# 644. "Impact of heat inactivation and freeze-thaw cycles on detection of influenza A and influenza B antigens and RNA when assessing novel multiplex diagnostic assays"

**DOI:** 10.1093/ofid/ofad500.708

**Published:** 2023-11-27

**Authors:** Anuradha Rao, Heather Bowers, Courtney Sabino, Kaleb McLendon, Evelyn Morales, Zianya Solis, Morgan Greenleaf, Julie Sullivan, Eric Lai, Gregory L Damhorst, Wilbur A Lam, Leda Bassit

**Affiliations:** Emory University School of Medicine,, Atlanta, Georgia; Emory University, Atlanta, Georgia; Emory University, Atlanta, Georgia; Emory University, Atlanta, Georgia; Emory University, Atlanta, Georgia; Emory University, Atlanta, Georgia; Emory University, Atlanta, Georgia; Emory University, Atlanta, Georgia; VentureWell, San Diego, California; Emory University, Atlanta, Georgia; Emory University School of Medicine/Georgia Institute of Technology, Atlanta, GA; Emory University, Atlanta, Georgia

## Abstract

**Background:**

Advances in diagnostics since the emergence of COVID-19 has resulted in easy availability of rapid antigen tests (RAT). Building on this, multiplex tests for simultaneous detection of Flu A, Flu B, and SARS-CoV-2 are being evaluated as part of NIH’s Rapid Acceleration of Diagnostics (RADx) program. Critically important for assay development and assessment are viral panels prepared under validated conditions to evaluate new tests. These panels may contain live virus or may be inactivated to promote biosafety. They also may undergo freeze-thaw cycles during storage and transport. We characterized the effect of heat inactivation (HI) on detection of Flu A and B using two RATs, and the effect of freeze-thaw cycles on a point-of-care (PoC) molecular assay already on the market.

**Methods:**

Panels were prepared by serially diluting Flu A or B strains obtained from BEI Resources. Both live and HI panels were prepared. HI was achieved in-house (60°C, 30 minutes). Panels with and without HI were used to assess two RATs by performing triplicate tests and determining limit of detection (LOD) as the lowest TCID50 at which 3 of 3 replicates were positive. Additionally, a molecular-based PoC assay was evaluated by recording cycle threshold (Ct) values before and after freeze-thaw of specimens. All assays were performed according to the manufacturer’s instructions.

**Results:**

Both RATs detected Flu A with similar sensitivity (2-fold increase in LOD) before and after heat inactivation (Figure 1), whereas Flu B was not detected by either RAT after heat inactivation. These results were consistent across both RATs and two strains of each virus. Live Flu A and B were stable and consistently detected up to four freeze-thaw cycles in the molecular assay with minor attrition of Ct values (Figure 2).

Results of LOD characterization for two independent RATs with influenza A and B with and without heat inactivation.

**Figure:**
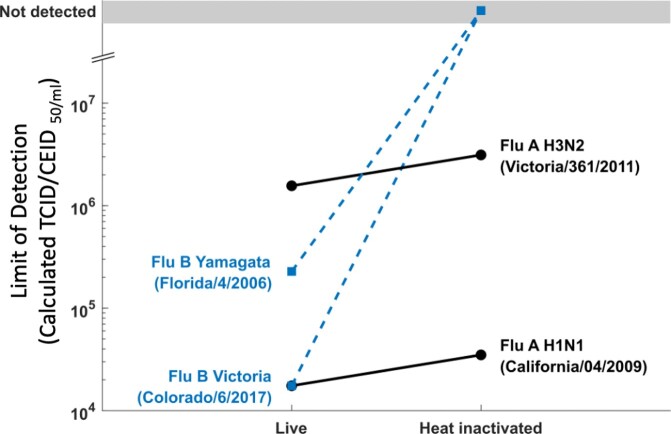

Correlation of Flu A and Flu B Ct values for fresh samples versus after four cycles of freeze thaw measured using a PoC molecular assay.

**Figure:**
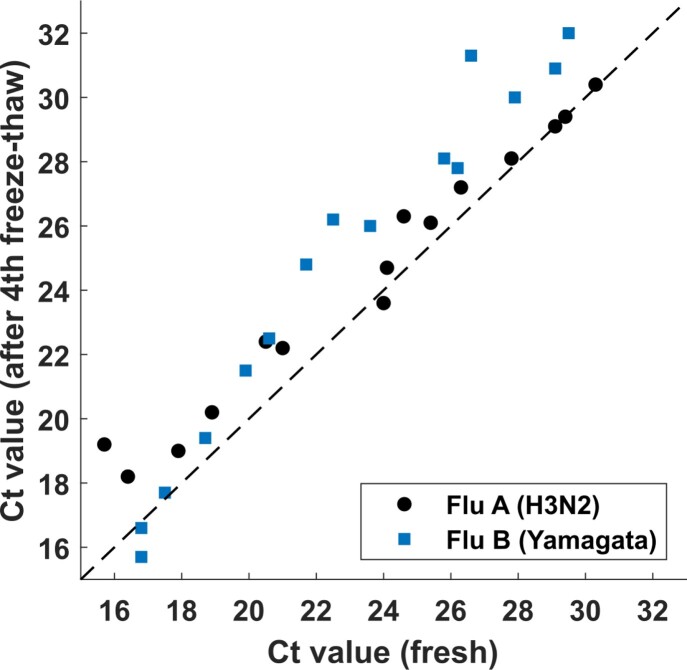

**Conclusion:**

Pre-treatment and storage of testing material plays a crucial role in the assessment of diagnostic tests used for Flu A and B detection. Loss of reactivity in Flu B antigen tests after inactivation suggests that heating may denature a critical epitope recognized by immunoassays. Selection of live versus HI samples and degradational effects of freeze thaw cycles must be considered by laboratorians and engineers creating or validating diagnostic tests.

**Disclosures:**

**All Authors**: No reported disclosures

